# The importance of viewshed in nest site selection of a ground-nesting shorebird

**DOI:** 10.1371/journal.pone.0319021

**Published:** 2025-02-20

**Authors:** Sharon S. Dorsey, Daniel H. Catlin, Shannon J. Ritter, Christy N. Wails, Samantha G. Robinson, Katie W. Oliver, Henrietta A. Bellman, Sarah M. Karpanty, James D. Fraser

**Affiliations:** Department of Fish and Wildlife Conservation, Virginia Polytechnic Institute and State University, Blacksburg, Virginia, United States of America; University of Fribourg, SWITZERLAND

## Abstract

Birds and their nests are vulnerable to predation during the breeding season. Many birds have evolved nest placement strategies that minimize risk such as concealing nests in vegetation, or nesting in inaccessible cavities or on cliffs. Some ground-nesting species choose open areas where vegetative concealment or physical protection is minimal. These species may benefit from the ability to visually detect predators approaching the nest, affording them more time to perform evasive or distracting behaviors. We studied the nesting behavior of piping plovers (*Charadrius melodus*) on Fire Island, New York from 2015–2020 to determine if the area visible from the nest (i.e., ‘viewshed’) affected nest site selection. We calculated viewsheds at nests and random points and evaluated nest site selection using logistic regression modelling. Piping plovers selected nest sites with a greater view of predators than would be expected if nest site selection was random relative to viewshed. The inclusion of viewshed improved the predictive ability of a previous nest site selection model that was based on habitat characteristics present on the landscape in 2015, but its influence weakened as ecological succession progressed. Topographic variation was the predominant visual obstruction source at plover nest sites compared to vegetation height. Viewshed may play a role in nest site selection in other ground-nesting birds, and thus is an important factor to consider in the development of habitat management strategies and in understanding the evolution of behavior.

## Introduction

The nesting period is a vulnerable stage of a bird’s life [1]. Nest survival often is lower than hatchling, fledgling, or adult survival [1–4]. To reproduce successfully, a bird and its offspring must survive the nesting period by avoiding potential threats such as those posed by the environment (e.g., inclement weather) [5–7] or other species (e.g., predators). Nests are especially vulnerable, as predators may access unattended eggs, or be attracted to them when parents go to and from the nest [8–10]. Significant nest failure can affect a species breeding population size [11].

Some nest protection strategies are a function of the nesting location itself (e.g., ground, elevated, cavity). Nesting in arboreal cavities or subterranean burrows provides protection from weather events (e.g., storms and wind) and moderates temperature fluctuations [12]. Additionally, cavity nests may be less vulnerable to predators than surface nests [13]. Nesting within vegetated canopies can provide shelter and reduce detection by predators [14,15]. Surface ground nesters, however, often rely on crypsis to avoid detection and behavioral cues from other animals to initiate predator deterrence to improve survival for themselves and their eggs or young [16]. Further, birds have evolved adaptations across nesting strategies to protect their nests and themselves during the nesting period [17,18]. For example, direct physical aggression (e.g., hovering, diving, and striking) is observed in arboreal-nesting hummingbirds (e.g., *Archilochus* sp.), ground-nesting terns (e.g., *Sterna* sp.), and cavity-nesting swallows (*Tachycineta* sp.) [19–21], and indirect displays or vocalizations directed at a threat are observed in cavity-nesting woodpeckers, canopy-nesting warblers, and ground-nesting shorebirds (*Charadrius* sp.) [22–24]. Additionally, distraction displays such as injury feigning (e.g., broken wing displays) have been recorded in numerous species, comprising 52 bird families with varying nest types, and are deployed as a defense against a variety of predators [25].

Many ground-nesting birds choose exposed sites in open sand, gravel, rocks, or short vegetation. Examples include shorebirds (Charadriiformes) [26,27], passerines (Passeriformes) [28], and waterfowl (Anseriformes) [29]. Cryptic coloration, stealthy nest visits, and distraction displays that lure predators away from nests or chicks may improve fitness of ground-nesting species [25,30]. These strategies may compensate for the danger of nesting in the open and constitute a trade-off between selecting a suitable microclimate and minimizing predation risk [30]. Nesting in exposed areas is common among shorebirds, and many nest on river, bay, or ocean shorelines. Shorelines are thin, often open areas between vegetated biomes and water, prone to occasional flooding or overwashing (the flow of water and sediment which can level subtle elevational gradients and suppress vegetative growth) [31,32], leaving open areas with little vegetation that are attractive to shorebirds.

An unobstructed view of the surroundings may be especially important for ground-nesting birds, as it could allow the bird on the nest to detect (visually and audibly) an approaching predator in time to inconspicuously depart the nest and successfully initiate a distraction display [25,33,34]. Natural and anthropogenic landscape features can affect a ground-nesting bird’s viewshed (visible area from a specified location) [35]. Viewshed likely is an important component of habitat selection and level of vigilance in animals [35–37]. Excluding one study by Gómez-Serrano and López-López (2014) that found viewshed at Kentish plover (*Charadrius alexandrius*) nests had greater visibility relative to control points, viewshed has not yet been formally tested as a factor in nest site selection models for any other ground-nesting species, due in part to the difficulty of estimating viewshed in a field setting [34,38–40]. However, applications of remote sensing offer a new approach to measure viewshed in wildlife habitats [35,41].

The piping plover (*Charadrius melodus*; hereafter ‘plover’) is an imperiled shorebird [42,43] that nests on sandy beaches and sandbars in North America. Obstruction of viewshed by vegetation may negatively affect plover nest site selection [38,40,44]. We investigated the hypothesis that plovers select nest sites with unobstructed viewsheds. We quantified the change in viewshed over time with vegetative growth and topographic development using remote sensing technology. Our objectives were to (1) test whether viewshed was greater at plover nests than at unoccupied random points, (2) determine if adding viewshed as a covariate improves the predictive ability of a previous model of nest site selection [45], (3) determine if the effects of viewshed changed through the course of ecological succession following a landscape-level disturbance event, and (4) quantify the relative contribution of vegetative succession and topographic development in obstructing the viewshed at plover nests.

## Methods

### Study species

The piping plover is a small, sand-colored, ground-nesting migratory shorebird inhabiting coastal, lacustrine, and riverine shorelines in the United States and Canada [46]. On the Atlantic Coast, birds return between mid-March and May from their southern non-wintering grounds to the northern breeding sites, stretching from North Carolina, USA to Newfoundland, Canada and begin establishing pairs [46]. The overall nesting period (i.e., at least one pair still incubating) on the Atlantic coast typically starts in early April and can extend to late July [47,48]. Plovers nest on open sand or gravel, where the bird’s sandy plumage provides cryptic protection from predators. When a predator is detected during the incubation period, parental plovers may perform several anti-predator behaviors, including a stealthy nest departure, feigning injury, false brooding, and vocal distractions to lure predators away from their nest site [49,50].

### Study area

**Fig 1:**
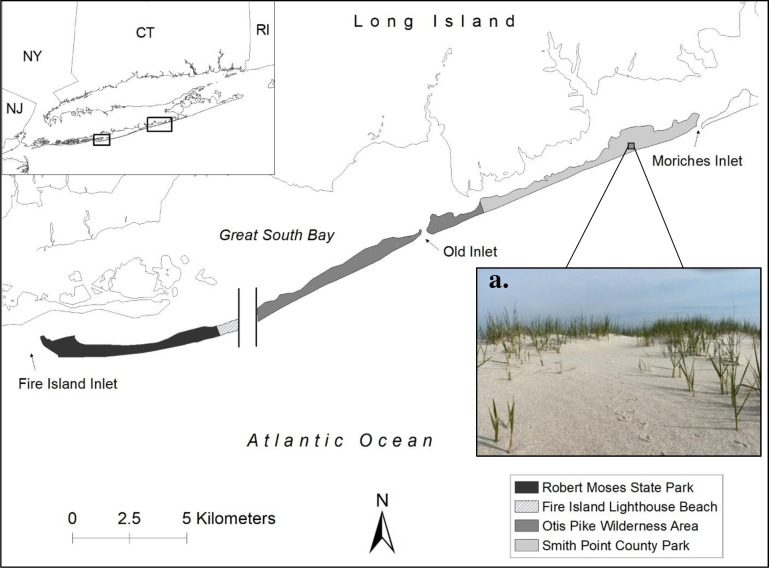
Map of study area.

We studied plovers on Fire Island, New York (40.710604°N, −72.931583°W; Fig 1). Fire Island is a narrow barrier island near the south shore of Long Island, NY. Fire Island is bounded by the Atlantic Ocean to the south, Fire Island Inlet to the west, the Great South Bay and several smaller bays to the north, and Moriches inlet to the east (Fig 1).

Map of the study area (gray) at Fire Island, NY. Fire Island is comprised of Robert Moses State Park (managed by New York State Parks), Fire Island Lighthouse Beach, Otis Pike Wilderness Area (both managed by the National Park Service (NPS), and Smith Point County Park (managed by Suffolk County Parks). The area between the vertical black lines comprised a 25-km long residential area excluded from the study, and thus was collapsed in the map. a) A photograph that illustrates the perspective of an adult plover facing a vegetated slope in their nesting habitat within Smith Point County Park beach on 14 April 2021. Made with Natural Earth. Free vector and raster map data @ naturalearthdata.com.

Main habitats included ocean-front sandy beaches, bayside intertidal flats, undulating dune systems, sparse maritime forests, and salt marshes [45,51]. Such coastal systems are dynamic, and frequent storms drive changes in vegetation coverage and island geomorphology [52,53]. In October 2012, storm surges from Hurricane Sandy reached the coastline of several barrier islands, breaching Fire Island in two locations [54]. Following Hurricane Sandy, from 2014–2016, the U.S. Army Corps of Engineers repaired one of the breaches, built dunes, widened parts of the beach, and planted American beachgrass (*Ammophila breviligulata*) to stabilize the shoreline [55]. The second breach occurred in a previous inlet location (Old Inlet) and remained untouched following the Hurricane due to its location in a National Park Service Wilderness Area (Fig 1).

Dominant vegetation in the area comprised American beachgrass (*Ammophila breviligulata*), common reed (*Phragmites australis*), seaside goldenrod (*Solidago sempervirens*), beach pea (*Lathyrus japonicus*), and wooly beach heather (*Hudsonia tomentosa*) [56]. The climate is temperate coastal with four distinct seasons. During the study, minimum temperatures were 4–25°C and maximum temperatures were 11–37°C.

### Terrestrial predator community

The most abundant terrestrial mammals at Fire Island included native red fox (*Vulpes vulpes*), native northern raccoon (*Procyon lotor*), and introduced domestic cat (*Felis catus*) [57–59], and each is a known or a likely predator of plovers or their nests [48,57,60,61]. Depredation accounted for approximately 35% of nest failures at this site [62]. Virginia opossum (*Didelphis virginiana*) also were present but were not seen visiting a plover nest [57]. The red fox population within the study area was carefully monitored throughout two outbreaks of sarcoptic mange (2015, 2017), until fox extirpation in 2019 [57,58]. Red foxes imposed an indirect threat (e.g., nest disturbance, fear), and their presence was likely correlated with predation [63]. Adult plover depredation by a cat was documented via fecal sample in 2018 [57] and plover nest failures were attributed to cat depredation at Fire Island in 2022 and 2023 [59], and on the adjacent Westhampton Island, NY [64].

### Field methods

#### Nest searching.

We surveyed beaches for plovers from April to August during 2015 – 2020. We searched for nests every 1–3 days by walking within potential dry sand nesting habitat and by observing plover behavior [45,51,65]. We recorded nest locations + /- 1m) using a Trimble Geo7x GPS unit (Trimble, Sunnyvale, CA, USA). We visited all nests every 1–3 days until they hatched or failed. We used nesting data collected by land managers in 2020 as the novel SARS-CoV-2 pandemic precluded our fieldwork. The managers followed similar protocols, but they visited less frequently than our crews.

#### Vegetation sampling.

We used vegetation height data recorded during the time of plover nest initiation at randomly selected points from April to June during 2016 (n = 16), 2017 (n = 30) [66], and 2022 (n = 55). We used ArcMap 10.5 (2016–2017; Esri, Inc., Redlands, CA, USA) to generate random points ( > 60 m apart) across the study area each year. Once we navigated to the point, we placed a 1-m quadrat on the ground, and if vegetation was present, we recorded the dominant plant species (greatest proportional cover) in the quadrat. We measured the maximum vegetation height ( ± 0.1 m) using a Robel Pole [66,67]. In 2022, we returned to all the same points where vegetation was recorded in 2016 or 2017 and re-sampled the vegetation within the 1-m quadrat, using the same methods.

### Data analyses

#### Imagery classification.

We obtained aerial imagery (15-cm resolution) and Light Detection and Ranging (LiDAR; 1-m resolution, 2 pt/m^2^) data collected by aircraft during the nest site selection period in April (2015–2020; Axis Geospatial LLC., Easton, MD, USA). We classified the imagery into four land cover classes (dry sand, wet sand, water, and vegetation) using the ‘Maximum Likelihood Classification’ tool in ArcGIS 10.6 (Esri, Inc.). We manually digitized and masked human infrastructure or development (e.g., parking lots, jetties, buildings) from the imagery [37,45,51]. We used LiDAR (NAVD 1988) to determine the annual ground elevation across the landscape. Since the timing of aerial imagery did not consistently align with low tide, we used LiDAR data to delineate intertidal zones. The ocean intertidal line was set to 0.00–1.20 meters above sea level (MASL) in elevation, calculated in reference to the highest astronomical tide at the NOAA Moriches Inlet tidal station. The bay intertidal zone was set to 0.00–0.44 MASL, calculated in reference to the mean high water measurements at the NOAA Smith Point Bridge tidal station [68].

#### Calculating viewshed and spatial variables.

**Fig 2:**
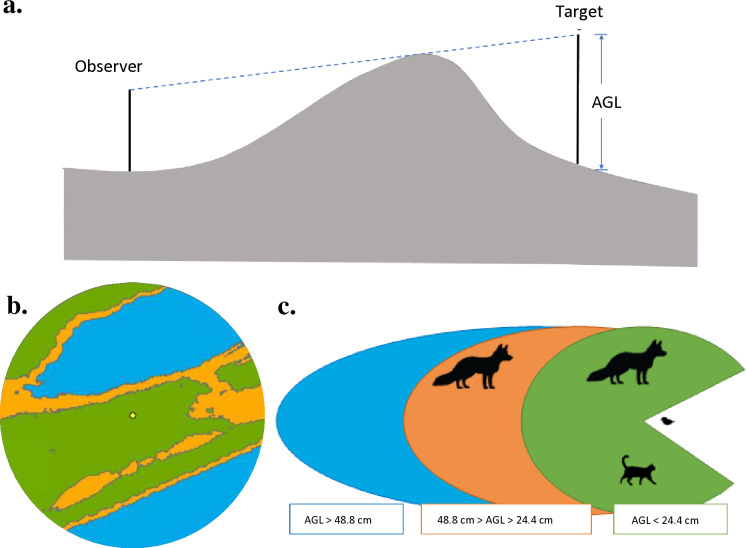
Viewshed explained. a) Conceptual diagram of the above ground level (AGL) setting demonstrating the height that a nonvisible target cell would need to be elevated by to become visible to the observer (figure adapted from Esri, Inc., Redlands, CA, USA). b) Example viewshed analysis output produced in ArcGIS Pro showing areas where all terrestrial predators (green), only large predators (orange), and no terrestrial predators (blue) would be visible within 30 m of an incubating plover (yellow circle). c) The above ground level (AGL) height ranges where specific predator classes would be visible to an incubating plover – AGL ≤ 24.4 cm (green) indicates where all terrestrial predators are visible to an incubating plover; AGL ≥ 24.4 and ≤ 48.8 cm AGL (orange) indicates where large predators are visible, but predators smaller than red fox are concealed by landscape features; AGL ≥ 48.8 cm (blue) indicates where no terrestrial predators are visible to an incubating plover.

**Fig 3:**
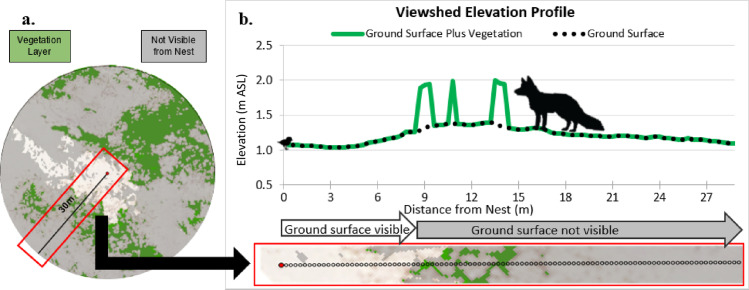
Example ArcGIS output of viewshed analysis within a 30-m buffer of the center point, illustrating areas visible and not visible due to vegetation height and elevation changes. (a) The map shows three key features: **gray** areas represent locations where visibility of predators is obstructed by either vegetation height or elevation changes, **green** areas represent vegetation cover, and **white** areas are visible from the nest site. This visibility (in **white**) is represented as a percentage of total visibility achievable within the 30-m diameter circle for the analysis. (b) Example cross-section profile showing the relationship between distance from the nest and visibility of predators. The plot indicates the distance at which a visual obstruction occurs in that particular direction of view. For example, no predator would be visible from the nest beyond 8 meters if obstructed. The visibility of a predator in the green areas depends on both the height of the vegetation and the height of the predator relative to the observer’s position at the nest.

We quantified the viewshed at nests and unoccupied, random points for 2015–2020. In each year, we generated four random points for every one nest using the ‘Create Random Points’ tool in ArcGIS Pro (Esri, Inc.). Random points were constrained to be within pixels classified as dry sand and > 30 m away from the nearest random point, corresponding to the mean flush initiation distance for two plovers in a comprehensive study [69]. We used the ‘Viewshed’ tool in ArcGIS Pro to classify pixels as visible or not visible from an observation point given visual obstructions surrounding it. We calculated viewshed from the perspective of an incubating plover’s eye height (6 cm) [46], and for the surrounding landscape we used ground elevation and classified vegetation (15-cm resolution). To estimate vegetation height, we used the sampled vegetation heights in a logarithmic regression to estimate annual median height for 2015–2020 and added those height values uniformly to the ground elevation for pixels that were classified as vegetation. We used the above-ground level settings within the ‘Viewshed’ tool to establish viewshed conditions based on an incubating plover’s ability to see predators. The above-ground level setting classifies each 15-cm pixel by whether an object ≥ a specified height would be seen by the plover given the landscape features (Figs 2 and 3).

We first examined a plover’s ability to view the full suite of common terrestrial predators of various sizes in the study area (i.e., red fox, northern raccoon, Virginia opossum, and domestic cat; ‘all predators’ viewshed), assuming that the ability to see the smallest predators (Virginia opossums, domestic cats) implies the ability to see taller predators as well. We used a cat’s average shoulder height (24.4 cm) [70] as the above ground level setting when quantifying the ‘all predators’ viewshed. We also examined a plover’s ability to view a red fox (‘large predators’ viewshed), the tallest common terrestrial predator (average shoulder height 48.8 cm) [71] and believed to be a key threat to Fire Island plovers [48,58]. We established the above ground level setting as 48.8 cm to quantify the large predators viewshed while predators < 48.8 cm in height would be obscured by landscape features. We restricted the viewshed analysis to a 30-m radius from each point based on the approximate flush distances of plovers (i.e., the distance between the bird and a perceived threat at which the bird leaves its nest) [38,49,72]. We report the area in which it would be possible for an incubating plover to see terrestrial predators (all predators, large predators) within the buffer as a percentage of the total 30-m buffer area (approximately 2,815 m^2^).

Walker et al. (2019) found six variables influenced plover nest site selection in 2015, after Hurricane Sandy, in a study area that overlapped with the one we considered here. We used ArcGIS to calculate the same six variables: Euclidean distance to bay intertidal zone (m), least cost distance to bay intertidal zone (m), least cost distance to ocean intertidal zone (m), elevation (m), distance to nearest development (m), and backshore beach width (m). Euclidean distance, calculated using the ‘Euclidean Distance’ tool, was the shortest distance from a point to an intertidal line. Least cost distance, calculated using the ‘Path Distance’ tool, was the shortest distance achievable by a plover walking on wet or dry sand (i.e., avoiding vegetation or open water). Backshore beach width was the distance perpendicular from the shoreline to the nearest back-beach barrier (i.e., vegetation or elevation change > 3 m) and calculated for every 10-m section of the study area using the ‘Create Fishnet’ tool. To directly compare with the methods employed by Walker et al. (2019), we computed values for the same six variables for the nests and random points in 2015, and both viewshed values.

#### Statistical analyses.

We examined whether the addition of the viewshed variable in the Walker et al. (2019) model made it more informative. We standardized (1 SD) all habitat variables in the dataset and constructed generalized linear models (GLMs) with a binomial error distribution and logit link. Our dependent variable represented used locations (1) and random points represented available locations (0; i.e., third order selection) [73,74]. We constructed these models in the R package ‘lme4’ [75]. We evaluated eight candidate models, all of which, except for the null model, were based on the Walker et al. (2019) model, including viewshed, years since Hurricane Sandy, and the interaction as additional factors. We produced models with all additive and interactive forms of viewshed and years since hurricane. We checked all standard model assumptions, measured fit of the models with a Hosmer and Lemeshow goodness-of-fit test [74], and ranked the eight candidate models using Akaike’s Information Criterion corrected for sample size bias (AIC_*c*_). The most parsimonious model (lowest AIC_*c*_) in the comparison was chosen as the top model if there was > 2 Δ AIC_*c*_ between the first and second ranked models [76,77]. To determine the overall influence of viewshed on nest site selection, we evaluated the top-ranked additive candidate model containing a viewshed variable and compared the ΔAIC_*c*_ values of models with large predator vs. all predators viewshed. We also assessed whether viewshed retained its importance in plover nest site selection over time in the years since storm (2015–2020), a period when vegetation succeeded and topography changed, by evaluating the beta coefficient of the interaction between the years since storm and the viewshed variable.

To determine the contribution of visual obstruction caused by topography as compared to vegetation at plover nests, we conducted another viewshed analysis in ArcGIS Pro using only ground elevation (i.e., no vegetation height added) to determine where the plover’s view was obstructed by topography. We then subtracted the results of the two viewshed analyses (i.e., vegetation and topography minus topography only) to isolate the contribution of each to a plover’s obstructed viewshed. We tested the difference in means of each obstruction type using a two-sample t-test (α = 0.05). All analyses were conducted in R ver. 4.1.1 [78].

### Ethics statement

Fieldwork protocols were approved by Virginia Tech’s Institutional Animal Care and Use Committee (protocols 14-003, 16-244, 19-248). Work was completed under the U.S. Geological Survey Federal Banding Permit #21446, U.S. Fish and Wildlife Service Endangered Species permit #TE-697823, New York State Department of Environmental Conservation endangered and threatened species license #314, U.S. Department of the Interior Scientific Research and Collecting permits (FIIS-2015-SCI-0011, FIIS-2016-SCI-003, FIIS-2017-SCI-004, FIIS-2018-SCI-004, FIIS-2022-SCI-0005), New York State Office of Parks, Recreation, and Historic Preservation permits (15-0700, 16-0393, 17-0755, 18-0168, 19-0128, 22-0040), and with approval by Suffolk County Parks.

## Results

### Viewshed

Median vegetation height varied by year and increased over time (2016: median height = 10 cm, IQR = 20 cm; 2017: median height = 20 cm, IQR = 20 cm; 2022: median height = 50 cm, IQR = 10 cm) and annual predicted vegetation heights increased from 2015 (15 cm) to 2020 (46 cm, Table 1). The ground elevation within nest buffers ranged from −0.69–11.06 MASL.

**Table 1:** Annual predicted vegetation height on Fire Island, New York based on sampled plots in 2016, 2017, and 2022.

Year	Years since storm^a^	Vegetation height (cm)
2015	3	15.0
2016	4	21.0
2017	5	28.0
2018	6	34.0
2019	7	40.0
2020	8	46.0

Dominant vegetation in the area comprised American beachgrass (*Ammophila breviligulata*), common reed (*Phragmites australis*), seaside goldenrod (*Solidago sempervirens*), beach pea (*Lathyrus japonicus*), and wooly beach heather (*Hudsonia tomentosa*). The predicted heights are based on a logarithmic regression of these samples.

^a^Hurricane Sandy occurred in 2012.

During 2015–2020, we recorded 353 nests and generated 1412 random points in the study area. Viewshed values ranged from completely open (100%) to nearly fully obstructed (0.03%) within the 30-m buffer (Table 2). Nest site visible area was greater in the years shortly after Hurricane Sandy and during beach stabilization (i.e., 2015–2016), but declined in later years (i.e., 2017–2020; Table 2). Most (73.4%) nest sites had at least 50% visible area to see any terrestrial predators. The large predators viewshed median value of percent visible area was always greater than the ‘all predators’ viewshed (Table 2). The visible area at nest sites and at random points became more similar over time (Table 2).

**Table 2:** Viewshed statistics for nests and random points.

		Nests					Random points				
			*Large Predator Viewshed*		*All Predators Viewshed*			*Large Predator Viewshed*		*All Predators Viewshed*	
Year	Years Since Storm^a^	n	Median Viewshed (%)	Visible Area Range	Median Viewshed (%)	Visible Area Range	n	Median Viewshed (%)	Visible Area Range	Median Viewshed (%)	Visible Area Range
2015	3	36	96.43 (21.23)	23.73–100.00	89.81 (25.70)	18.01–100.00	144	69.52 (48.29)	3.65–100.00	53.77 (49.24)	2.61–100.00
2016	4	37	93.48 (30.88)	38.73–100.00	81.72 (38.50)	25.81–100.00	148	69.92 (44.60)	0.77–100.00	55.51 (43.50)	0.36 –100.00
2017	5	51	76.60 (30.89)	17.31–100.00	64.19 (35.49)	7.30–97.92	204	75.88 (45.80)	10.39–100.00	63.00 (43.11)	7.10–100.00
2018	6	63	81.31(30.48)	7.01–100.00	69.79 (37.01)	4.51–100.00	252	64.91 (42.44)	0.18–100.00	54.04 (43.41)	0.09–100.00
2019	7	92	74.83 (33.36)	13.60–100.00	64.82 (39.49)	9.81–100.00	368	74.60 (45.00)	0.35–100.00	63.48 (48.65)	0.19–100.00
2020	8	74	75.49 (32.60)	0.99–100.00	62.41 (35.20)	0.67–99.99	296	63.61 (50.42)	0.06–100.00	50.36 (49.10)	0.03–100.00
**All years**	**--**	**353**	**81.67(32.96)**	**0.99–100.00**	**69.17 (39.54)**	**0.67–100.00**	**1412**	**68.65 (46.53)**	**0.06–100.00**	**57.25 (47.91)**	**0.03–100.00**

Large predator viewshed (i.e., where a red fox is visible within 30-m buffer) and all predators viewshed (i.e., where all terrestrial predators are visible within 30-m buffer) for plover nests and random points in Fire Island, New York 2015–2020.

^a^Hurricane Sandy occurred in 2012.

Incorporating the viewshed variables improved the Walker et al. (2019) model for plover nest site selection. All models including viewshed were ranked higher (lower ΔAIC_*c*_) than the models without viewshed (Table 3). The model accounting for the interaction between the ‘large predators’ viewshed and years since storm was the most informative model (ω = 0.85; Table 3). Plovers selected nest sites with greater large predators viewshed (β = 1.26, SE = 0.31, 95% CI = 0.66 – 1.88; Table 4), but, as the time since Hurricane Sandy increased, the effect of viewshed in nest site selection lessened (β = −0.13; SE = 0.05; 95% CI = −0.23–−0.04; Table 4).

**Table 3:** Model selection statistics.

Model^a^	*k* ^b^	Δ AIC_*c*_	ω^c^	LL^d^
Walker (2019) + (Large predators viewshed × Years since storm)	10	0.00	0.85	−787.06
Walker (2019) + (All predators viewshed × Years since storm)	10	4.59	0.09	−789.35
Walker (2019) + Large predators viewshed + Years since storm	9	5.52	0.05	−790.83
Walker (2019) + Large predators viewshed	8	8.78	0.01	−793.47
Walker (2019) + All predators viewshed + Years since storm	9	12.35	0.00	−794.25
Walker (2019) + All predators viewshed	8	15.13	0.00	−796.65
Walker (2019)	7	37.49	0.00	−808.83
Null	1	174.18	0.00	−883.21

Candidate generalized linear models (binomial error distribution, logit link) including previously tested variables from Walker et al. (2019) [Distance to bay + Distance to development + Least cost distance to bay + Least cost distance to ocean + Elevation + Backshore beach width], viewshed, and years since storm (i.e., Hurricane Sandy) ranked by Akaike’s information criterion corrected for small sample sizes (AIC_*c*_) with predator class-based viewshed conditions comparing nest sites selected by piping plovers (*Charadrius melodus*) to random points in Fire Island, NY in 2015–2020.

^a^Model covariate large predators viewshed is defined as 48.8 cm above ground level; all predators viewshed is defined as 24.4 cm above ground level; Years since storm = 2015 is 3 years post-storm, etc.; Walker et al. 2019 variables include: Distance to bay, Distance to development, Least cost distance to bay, Least cost distance to ocean, Elevation, and Backshore beach width.

^b^Number of parameters.

^c^Model weight.

^d^Log Likelihood.

**Table 4:** Covariate statistics from top-ranked habitat selection model.

Covariate^a^	β	SE	Lower 95% CI	Upper 95% CI
Intercept	−2.35	0.29	−2.91	−1.77
Years since storm	0.11	0.04	0.03	0.20
Large predators viewshed	1.26	0.31	0.66	1.88
Distance to bay	−0.24	0.07	−0.39	−0.10
Distance to development	0.24	0.06	0.12	0.37
Least cost distance to bay	−0.37	0.09	−0.55	−0.21
Least cost distance to ocean	−0.16	0.08	−0.31	−0.02
Elevation	−0.14	0.08	−0.30	0.03
Backshore beach width	0.32	0.06	0.21	0.44
Years since storm × Large predators viewshed	−0.13	0.05	−0.23	−0.04

Standardized estimates (β), standard errors (SE), and lower and upper 95% CIs for covariates for the top-ranked generalized linear model (binomial error distribution, logit link) comparing nest sites selected by piping plovers (*Charadrius melodus*) to random points in Fire Island, New York in 2015–2020.

^a^Covariates are defined as: Years since storm = 2015 is 3 years post-storm, etc., and large predators viewshed = 48.8 cm above ground level.

### Visual obstruction sources

The contribution of vegetation height and topography to visual obstruction at nests fluctuated annually (Table 5). Most (77%) of the visual obstruction within a nest site viewshed was caused by topographical (elevation) variation. Across all years of our investigation, vegetation obstructed a plover’s view of larger predators marginally (median = 0.21%, IQR = 3.54%; Table 5); however, topography obstructed the view (median = 13.7%, IQR = 28.4%; Table 5) to a greater degree than vegetation height (t = −12.64; df = 704; p < 0.001).

**Table 5:** Percent of visual obstruction at nests.

Year	Years Since Storm^a^	Median Vegetation Obstruction Area (IQR)	Median Topography Obstruction Area (IQR)
2015	3	0.00 (0.00)	3.52 (21.23)
2016	4	0.07 (1.63)	6.01 (17.58)
2017	5	0.73 (1.91)	19.69 (31.33)
2018	6	0.02 (2.16)	12.56 (25.75)
2019	7	1.56 (6.37)	16.57 (29.56)
2020	8	0.47 (7.01)	17.39 (25.53)
All Years	**--**	0.21 (3.54)	13.73 (28.41)

Median area (%) and interquartile range (%) of visual obstruction caused by vegetation or topography within the 30-m nest buffer at piping plover (*Charadrius melodus*) nests on Fire Island, NY in 2015–2020. Values are derived from the large predators viewshed (i.e., where a red fox is visible within 30-m buffer).

^a^Hurricane Sandy occurred in 2012.

## Discussion

### Viewshed’s role in nest site selection

Viewshed was important in plover nest site selection and provides a way to compare visibility across sites. Two passerine species preferred higher visibility sites over more obstructed ones [79,80] and our study agrees with these past findings. Staying vigilant about the threats from predators is essential for the survival of prey species. It has long been asserted that plovers favor open nesting areas [38,81,82]. Burger (1987) emphasized that plovers’ selection for open areas increases the potential for predator detection. Despite this, quantitative evidence about nest site selection was lacking. In a meta-analysis of 53 studies of predation on bird’s nests in multiple habitats in North America, 24% of nests were lost to terrestrial predators, of which 63% were canids, northern raccoons, and Virginia opossums [10]. Thus, our study has broad implications.

The line of sight between predator and prey is a function of predator height, distance to one another, and landscape features (e.g., vegetation height and microtopography; Andersson et al. 2009). When the predator is not easily detectable, or visible, a bird is obviously less likely to spot the predator itself [83]. Predation risk of its nest and itself may then increase because the parent must detect the predator before realizing the need to take evasive action [84]. Thus wariness, defense, and flight have coevolved to form a bird’s behavior regarding nest defense. We evaluated conditions where known terrestrial predators would be within a plover’s line of sight given predators’ heights and the landscape features. Although both viewshed conditions predicted nest site selection better than the null model, the ‘large predators’ (e.g., red fox) viewshed was a stronger indicator than the ‘all predators’ (e.g., domestic cats) viewshed, suggesting that plovers selected nest sites that allowed for detection of large, not small predators. Cats are, however, a significant threat to nests, chicks, and adults (C. Wails, unpublished data), for which, piping plovers may be ill-equipped if they overlook smaller predators.

As the predator community and land cover changed, the effect of predator viewshed on plover nest site selection seemed to decline. There are several possibilities for viewshed’s shift in importance. First, plovers may be prioritizing other nest site variables (e.g., avoiding flood-prone low elevation areas) in a way that compromises the visibility at nest sites. Similarly, Oriental storks (*Ciconia boyciana*) demonstrate compromising adaptive nesting strategies by nesting further from food sources in areas of higher human disturbance, prioritizing nest protection over resources access [85]. Second, as plover population density rose in a heterogenous habitat, the competition for nests with ample visibility intensified, forcing some plovers into visually obscured nesting sites [65,86,87]. A study on European shags (*Phalacrocorax aristotelis*) revealed an inverse relationship between population size and the average suitability of nest sites occupied [88]. Third, the extirpation of the plover’s primary predator, the red fox, could have reduced the importance of viewshed to detect fox in the succeeding years (i.e., predator-induced plasticity) [89]. The decline of terrestrial predator abundance and emergence of aerial predators reliant on visual search could signal a switch in the roles of cover and camouflage in plover nest site selection [40], where predator avoidance becomes the priority instead of predator detection. And finally, plovers exhibit high nest site fidelity [90], which could obscure habitat selection relative to vegetation cover, which increased during the study. Plovers on the Missouri River chose to nest at the foot of cottonwood saplings, but as the seasons progressed, emerging leaves made the habitat seem unsuitable [91].

### Visual obstruction

Vegetation has been widely believed to be the primary source of limiting visibility in ground-nesting birds [49,81,82,92–94]. However, in this study, topography, not vegetation height, was the primary source of visual obstruction to an incubating plover. Microtopography (e.g., footprints, small depressions, and dunelettes) as a type of visual obstruction to ground-nesting birds is little studied, although it may obstruct predator-prey visibility and impede visibility of other individuals in a shared environment [36,95,96,97]. Larger topographical features, like beach dunes, are obvious view-obstructing features [98]. Beach dunes often appear in shorebird habitat selection studies as distance from dune to nest [38,99,100], in terms of nest elevation [87,101,102], or a nesting habitat type [99]. Nesting in low elevations reduces predator visibility but increases vulnerability to depredation and flooding, and elevated sites enhance visibility but risk exposure to wind-driven sand abrasion and depredation [38]. Over the course of ecological succession, vegetated dune systems developed across the nesting habitat through a positive feedback loop between sand deposition and plant colonization [103,104]. We noted that both vegetation and topographical obstruction within the nest sites increased over time, thus the development of topographical obstruction would be expected without an event similar to Hurricane Sandy or another high water event.

## Conclusion

As technology advances, our understanding of habitat selection advances, too. Image quality and availability, coupled with computational power, facilitated our analysis of viewshed. Viewshed as measured by modern techniques can provide insight into how birds select habitats. This study contributes to nascent viewshed techniques investigating animal behavior [35,105,106], and it is the first conducted for ground-nesting shorebird species or performed with such fine-scale geospatial layers, allowing for the three-dimensional visualization. Importantly, this geospatial approach offers an innovative and consistent method for viewshed assessment in other ground-nesting species, as it can be adapted for other birds of conservation concern, such as the red knot (*Calidris canutus*), bar-tailed godwit (*Limosa lapponica*), and mountain plover (*Charadrius montanus*). It should be mentioned that nest site selection does not necessarily mean improved nesting success. Thus, studies on the effect of viewshed on reproductive output are needed. Further investigation into overlooked factors shaping nest-site selection choices using new approaches, as we did with viewshed, allows for more informed and tailored conservation strategies. We suggest that viewshed can be an important component to nest site selection, and thus could help determine the habitat needs of species such as the piping plover.
